# Prospective Transcriptomic Pathway Analysis of Human Lymphatic Vascular Insufficiency: Identification and Validation of a Circulating Biomarker Panel

**DOI:** 10.1371/journal.pone.0052021

**Published:** 2012-12-18

**Authors:** Shin Lin, Jeanna Kim, Mi-Joung Lee, Leslie Roche, Nancy L. Yang, Philip S. Tsao, Stanley G. Rockson

**Affiliations:** Stanford Center for Lymphatic and Venous Disorders, Division of Cardiovascular Medicine, Stanford University School of Medicine, Stanford, California, United States of America; University of Nebraska Medical Center, United States of America

## Abstract

**Background:**

In our previous transcriptional profiling of a murine model, we have identified a remarkably small number of specific pathways with altered expression in lymphedema. In this investigation, we utilized microarray-based transcriptomics of human skin for an unbiased *a priori* prospective candidate identification, with subsequent validation of these candidates through direct serum assay. The resulting multi-analyte biomarker panel sensitively should sensitively discriminate human lymphedema subjects from normal individuals.

**Methods and Findings:**

We enrolled 63 lymphedema subjects and 27 normals in our attempt to discover protein analytes that can distinguish diseased individuals from controls. To minimize technical and biologically irrelevant variation, we first identified potential candidates by performing transcriptional microarray analysis on paired diseased and normal skin specimens sampled from the same individuals. We focused our attention on genes with corresponding protein products that are secreted and took these candidates forward to a protein multiplex assay applied to diseased and normal subjects. We developed a logistic regression-based model on an eventual group of six proteins and validated our system on a separate cohort of study subjects. The area under the receiver operating characteristic curve was calculated to be 0.87 (95% CI : 0.75 to 0.97).

**Conclusions:**

We have developed an accurate bioassay utilizing proteins representing four central pathogenetic modalities of the disease: lymphangiogenesis, inflammation, fibrosis, and lipid metabolism, suggesting that these proteins are directly related to the pathogenesis of the tissue pathology in lymphatic vascular insufficiency. Further studies are warranted to determine whether this newly-identified biomarker panel will possess utility as an instrument for *in vitro* diagnosis of early and latent disease; the ultimate applicability to risk stratification, quantitation of disease burden, and response to therapy can easily be envisioned.

## Introduction

Given the central role of the lymphatic system in circulatory, metabolic, and immune-related homeostasis, it is not surprising that lymphatic pathology is responsible for predictably broad and severe clinical sequelae. It is commonly accepted that lymphatic disease has an impact on the health and well-being of more than 1.3 million individuals throughout the world [1]. These chronic, debilitating lymphatic diseases are frequently misdiagnosed and under-recognized.

The complex pathological biology of lymphedema includes a disruption of normal cutaneous cellular architecture accompanied by a blunting of normal immune traffic and by the presence of a profound chronic inflammatory response in the tissues [2], [3]. Furthermore, lymphedema is uniquely characterized by the presence of adipocyte proliferation and hypertrophy, and progressive tissue fibrosis [4].

The pathogenesis of these complex tissue responses is only now beginning to lead to focused investigation. The biology of the lymphatic vasculature has, until quite recently [5], eluded substantial molecular scrutiny; thus, the diseases of this important circulation are still poorly understand and lack sophisticated modalities for diagnosis, risk stratification, and treatment. When fully expressed, the clinical presentation of lymphedema is not difficult to identify. However, the often-protracted latency of structural and functional defects continues to pose distinct challenges for risk stratification and for early diagnosis and intervention.

In 2008, the National Institutes of Health convened a Working Group to address these inequities. Among its seven discrete recommendations, this panel concluded that the identification of a suitable panel of biomarkers for lymphatic disease is essential both for the process of scientific investigation and for the clinical management of these vascular disorders [6].

In order to provide the investigative context for the development of a biomarker platform relevant to human lymphedema, our prior investigations have centered upon a murine tail model of acquired post-surgical lymphedema. This model is intended to simulate the cutaneous responses of human breast cancer lymphedema [2], [7], [8]. In order to elucidate the molecular identity of the cutaneous tissue response to lymphatic vascular insufficiency, we have previously undertaken large scale transcriptional profiling of the lymphedematous skin and soft tissues, utilizing a comprehensive mouse cDNA microarray and have thereby identified a relatively restricted list of ∼600 genes, and a small number of specific pathways, whose expression patterns distinguish the abnormal tissue biology that accompanies the interruption of normal lymphatic function in the skin [9], [10].

The remarkable structural and functional similarities between the experimental model and the human disease suggest that application of a parallel approach in human lymphedema (genome-wide transcriptional profiling of cutaneous, disease-specific expression patterns as a discovery tool for clinically relevant circulating biomarkers) should facilitate the development of focused and pragmatic cellular and molecular insights into the biology of human lymphedema. In this investigation, we utilized microarray-based transcriptomics of human skin to prospectively identify candidate secreted proteins (potential circulating biomarkers); validation of these candidates through direct serum assay has allowed us to develop a multi-analyte biomarker panel that sensitively discriminates human lymphedema subjects from normals.

## Methods

### Study Subjects for Cutaneous Punch Biopsy

All participants in this study gave informed written consent under a protocol approved by the Stanford University Institutional Review Board. Initial study subjects with lymphedema (n = 27) were recruited from the patient population of the Stanford Center for Lymphatic and Venous Disorders. Patients with either unilateral or bilateral lymphedema were eligible for inclusion. In unilateral edema, criteria included the history and physical examination compatible with a clinical diagnosis of chronic lymphedema, accompanied by the presence of a limb volume ≥110% of the contralateral normal limb *and*/*or* abnormal bioimpedance spectroscopy. For subjects with bilateral disease, the indicated history and physical findings were utilized to establish the clinical diagnosis of chronic lymphedema. In all cases, it was necessary for the subjects to have completed at least one course of complete decongestive lymphatic therapy and to maintain all chronic maintenance measures from the time of initial enrollment until the completion of all clinical events in the study.

### Study Subjects for Serum Analysis of Circulating Biomarkers

A second cohort of lymphedema subjects (n = 36) was recruited for prospective measurement of circulating protein biomarkers. The lymphedema cohort was recruited in an analogous manner to those already described. Normal healthy adult volunteers (n = 27) were utilized as control subjects; in all cases, a careful history and physical examination were utilized to exclude the possible presence of lymphatic vascular insufficiency in these subjects.

### Clinical Study Methodology

***Limb volume*** was quantitated through serial measurement, at 4 cm intervals, of the circumference of the limb along its long axis [11]. Measurements were performed with gauged tape to ensure a uniform stretching force. The volume of the limb was calculated through the application of the truncated cone formula [11]. In unilateral edema, the volume of the affected limb was expressed as a ratio of the volume of the contralateral limb.

***Bioimpedance spectroscopy*** was performed with the Impedimed SFB7 in all subjects with unilateral lymphedema. A four-electrode configuration was used. Measurements were related to the extracellular and intracellular fluid contents of the limb as previously described [12]–[14].

***Cutaneous punch biopsy*** Two contiguous 6 mm full-thickness punch biopsy specimens were obtained from the medial aspect of the forearm or calf, respectively, of the affected extremity with an Acu-Punch (Ft. Lauderdale, FL) disposable device. Comparable specimens were obtained from the contralateral normal limb. In bilateral lymphedema patients, normal skin was obtained from a biopsy site from one of the unaffected extremities. Biopsy specimens were immediately placed in formalin and TRIzol solution (Invitrogen, Catalogue # 10296-010), respectively. Following biopsy, the skin edges were sutured with a single butterfly suture, and the patient received a prophylactic antibiotic regimen of cephalexin 250 mg in 3 divided doses for each of two days, or an equivalent regimen. Punch biopsy was performed at the time of enrollment.

***Phlebotomy*** was performed prior to cutaneous biopsy in the standard fashion, using a small gauge needle inserted into the brachiocephalic vein. 10 cc of blood were withdrawn, and the serum was frozen at −80°C for subsequent multimarker analysis.

### Histology

Staining with hematoxylin-eosin (H&E) and anti-LYVE-1 antibody (LYVE-1) was performed on all biopsy specimens, as previously described [10].

### RNA Sampling and Extraction from Human Skin Biopsy Specimens

After tissue was harvested for histological examination, the remaining biological material was subjected to RNA isolation. Homogenates of whole skin were collected in 1 ml TRIzol for long term storage at −80°C until use. Total RNA was isolated according to the TRIzol protocol, using the manufacturer’s instructions The total RNA yield was assessed using the Thermo Scientific NanoDrop 1000 micro-volume spectrophotometer (absorbance at 260 nm and the ratio of 260/280 and 260/230). The total RNA integrity was assessed using the Agilent’s Bioanalyzer NANO Lab-on-a-Chip instrument (Agilent, Catalogue # G2940CA).

### Microarray Processing and Analysis

Cy3 and Cy5 florescent dyes labeled, amplified antisense complementary RNA (cRNA) targets were prepared respectively using the Agilent QuickAmp Labeling Kit (5190-0444) with 200 ng of input total RNA from each sample Labeled cRNA (850 mcg) was hybridized overnight to the Agilent Whole Human Genome 4 × 44 K slide (Cat. #G4845A) that contains 44,000 probes, including 19,596 Entrez Gene RNAs. The combined Cy3 and Cy5 fluorescent labeled cRNA were mixed with the blocking buffer, fragmentation buffer, and hybridization buffer then hybridized onto the Agilent’s 4×44 k whole genome microarray (G2519F) slide in a hybridization oven at 65C for 17 hours. The arrays slide was then washed and scanned on the Agilent microarray scanner following the manufacturer’s protocols. Data were extracted using Agilent Feature Extraction Software. The microarray data is available in the Gene Expression Omnibus database under accession GSE39477.

### Luminex Bead Assays

The custom human 51-plex kits were purchased from Affymetrix (Santa Clara, CA) and utilized according to the manufacturer’s recommendations with modifications as described below. Briefly, serum samples were mixed with antibody-linked polystyrene beads on 96-well filter-bottom plates and incubated at room temperature for 2 h followed by overnight incubation at 4°C. Plates were vacuum-filtered and washed twice with PBS+0.2% Tween-20, followed by incubation with biotinylated detection antibody for 2 h at room temperature. Samples were then filtered and washed twice as above and re-suspended in streptavidin-PE. After incubation for 40 minutes at room temperature, two additional vacuum washes were performed, and the samples re-suspended in Reading Buffer. Each sample was measured in duplicate. Plates were read using a Luminex 200 instrument with a lower bound of 100 beads per sample per analyte.

### Data Analysis

Microarray analyses were performed using the statistical program R [15]. Array quality was assessed using library arrayQuality [16]. Pre-processing was performed with library marray [17]. Library limma [18] was used to identify differentially expressed genes. Expression pathways were analyzed with GeneSpring [19]. Further statistical and bioinformatic analyses were performed with the Ingenuity Pathway Analysis (IPA, Ingenuity Systems, Inc.) software, the DAVID Bioinformatic Database (http://david.abcc.ncifcrf.gov) and the KEGG database (www.genome.jp/kegg/).

The data from the Luminex assays were normalized by calculating the z-score for each protein/plate. Logistic regression with L1-regularization and area under the curve (AUC) of the receiver operating characteristic (ROC) curve were performed using program LIBLINEAR [20]. ROC curves were plotted using library ROCR [21]. Confidence intervals of the AUC were calculated by with library pROC [22].

## Results

### Demographic Characteristics of the Enrolled Lymphedema Study Population

**Figure 1:**
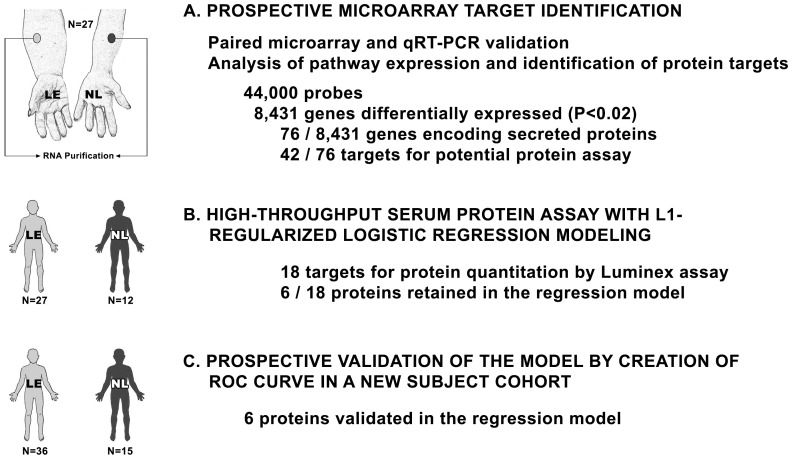
Schematic of the study design. A. Paired microarray of normal and lymphedematous skin derived from 27 subjects with lymphedema of one or more limbs. RNA was isolated from the whole tissue. Pathway analysis was performed to identify the final targets for protein analysis. B. High throughput assay of identified targets was performed using the Luminex 51-plex bead assay. Assays were performed on the original 27 lymphedema subjects and on 12 healthy normal controls. Logistic regression modeling was performed to identify the final targets for prospective analysis. C. Prospective assay for the 6 target proteins was performed on a distinct cohort of 36 lymphedema subjects and 15 normal controls. The results were analyzed through plotting a receiver operating characteristic (ROC) curve.

**Figure 2:**
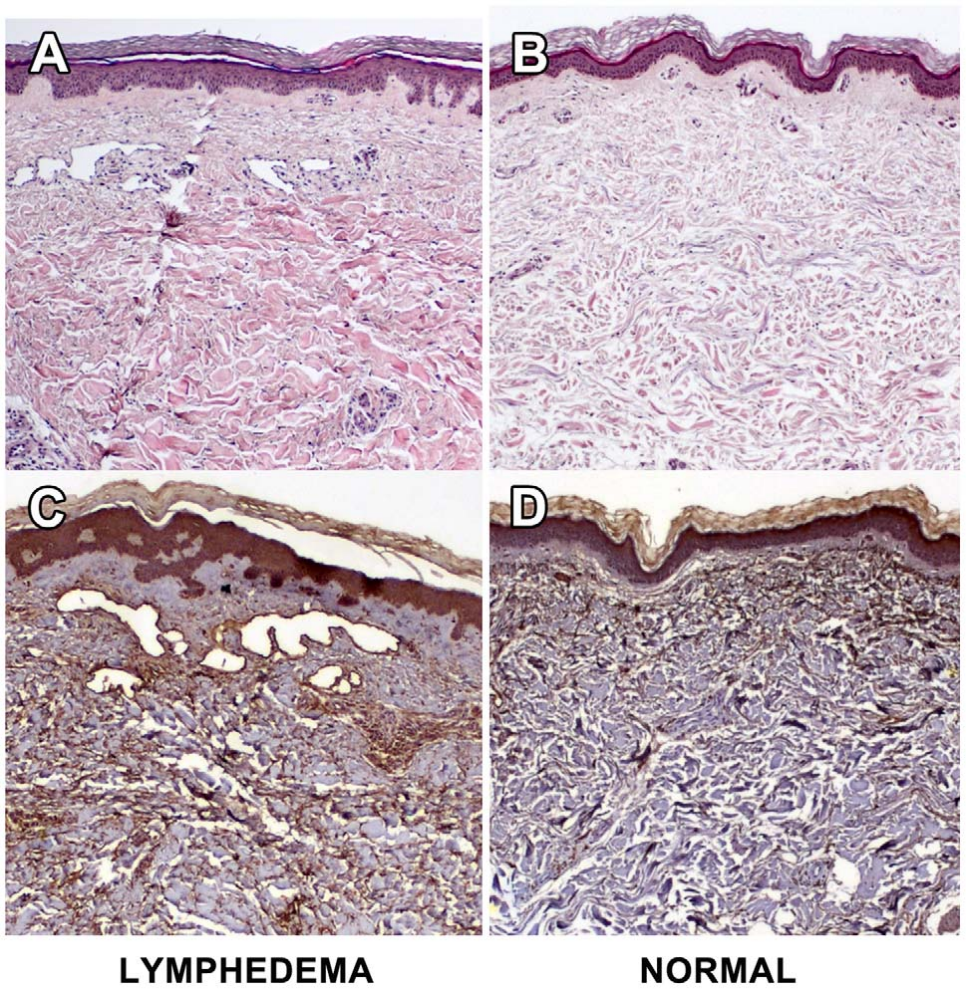
Representative histological findings in paired biopsy specimens of lymphedematous and normal skin. The specimens are derived from a representative study subject and reflect paired specimens from lymphedematous and normal limbs. A. Lymphedema skin H&E staining demonstrates an overall increase in the cellularity of the specimen that is particularly prominent in the epidermis and dermal-epidermal junction. There are prominent perivascular inflammatory infiltrates and there is obliteration of the dermis by dense eosinophilic material. Numerous dilated microvascular structures are seen in the upper dermis. B. Normal skin H&E shows normal cellularity, absence of inflammation and no notable microvascular changes. C. Lymphedema skin LYVE-1 staining demonstrates that the endothelial-lined microvascular structures seen on standard histology are lymphatic. There is evidence of positive microvascular lymphatic remodeling, as we have previously noted in the murine experimental model [10], [23], with an increase in the size and number of identified LYVE-1-positive structures. D. Normal skin LYVE-1 staining discloses scant-to-absent lymphatic structures in the dermis.

Our goal was to identify relevant protein biomarkers for lymphedema. The investigative approach is outlined in Figure 1. Our first task was to enroll subjects verified by a rigorous, uniform standard. For each patient, samples of normal and lymphedematous skin were procured by cutaneous punch biopsy. Cases required limb swelling upon visual inspection with quantitative documentation of pathological increases in limb volume, as well as objective measures that included abnormal bioimpedance spectroscopy and/or histological confirmation of the cutaneous pathology of lymphedema (increased cellularity, thickening and obliteration of the dermis, inflammatory infiltrates, and immunohistochemical documentation of positive microvascular lymphatic remodeling [23], [24]. Representative histology of diseased and normal skin specimens from the same patient are shown in Figure 2.

In total, 63 lymphedema study subjects were enrolled for study. The mean age was 56±13 years (range, 25–85 years), and 82% were female. Mean body mass index (BMI) for the lymphedema cohort was 28.48±7.01. Lymphedema duration was 13±10 years (range 2–51 years). The study cohort encompassed both primary and secondary lymphedema across a broad spectrum of etiologies (Table 1). Twenty-seven percent had arm involvement, and 73% had involvement of the leg(s). Twenty-two percent of the study subjects had bilateral disease.

**Table 1:** Lymphedema etiology.

Diagnosis	%
Primary Lymphedema	9.8
Venous Insufficiency	4.9
May-Thurner Syndrome	3.2
Breast Cancer	29.5
Cervical Cancer	11.5
Ovarian Cancer	3.2
Uterine Cancer	3.2
Hodgkin’s Lymphoma	3.2
Melanoma	1.6
Penile Cancer	1.6
Trauma	8.2
Infection	8.2
Other	11.5

### Prospective Transcriptomic Identification of Pathways and Circulating Biomarkers for Lymphatic Disease

In order to provide a discovery platform for the protein analytes to be characterized, we first performed transcriptomics to arrive at a set of candidates. Because cases and controls can never be perfectly matched, we chose to use normal and diseased skin from the same patient for each paired analysis, to decrease technical and biologically irrelevant noise. From an initial cohort of 27 lymphedema patients, we isolated whole tissue RNA and performed competitive microarray analysis. After whole tissue RNA isolation and purification from lymphedema and normal skin, 27 paired microarray analyses were performed. Of the 44,000 probes interrogated, 8431 genes were found to be differentially expressed at the pre-determined significance level of p≤0.02, in a paired manner between lymphedematous and normal samples. 1309 genes had differential expression at a significance of p≤0.001, and 313, p≤10^−5^.

### Analysis of Prospectively Identified Candidates

Analysis of the observed differential gene expression in lymphedema identified several significant pathways. Analysis by GeneSpring disclosed three significant pathways: transforming growth factor beta receptor [TGFBR] (p = 0.0002); epidermal growth factor receptor 1 [EGFR1] (p = 0.009); and interleukin 6 [IL6] (P = 0.002). These identified pathways underscore several of the hallmark biological features of chronic lymphedema, namely, fibrosis, dermal and epidermal cellular overgrowth, and inflammation [10].

Additional pathway analysis was performed through the KEGG (www.genome.jp/kegg/) database (Table 2). Of the 59 pathways identified, 13 were felt to have a direct mechanistic link to the expression of lymphedema in the skin: antigen processing and presentation (p = 2×10^−4^); insulin signaling (p = 8×10^−4^); graft-versus-host disease (p<0.004); adherens junction (p = 0.007); apoptosis (p = 0.02); adipocytokine signaling (p = 0.02); VEGF signaling (p = 0.04); Wnt signaling (p<0.05); PPAR signaling (p<0.07); natural killer cell mediated cytotoxicity (P = 0.08); T cell receptor signaling (p<0.09) and hedgehog signaling (p<0.10). The latter pathways additionally underscore the importance to the chronic disease state of alterations in cutaneous adipose biology, with implication of the insulin signaling pathway, and of (lymph)angiogenic signaling. The 300 differentially-expressed genes with the lowest observed p-values are listed in Table S1.

**Table 2:** Pathway analysis of differentially expressed genes by microarray.

TERM	COUNT	%	UNCOR-RECTED P-VALUE	FOLD EN-RICH-MENT	FDR
Prostate cancer	61	0.761	6.23E−06	1.56	0.008
Endometrial cancer	38	0.474	6.53E−05	1.66	0.082
**Antigen processing and presentation**	54	0.673	1.97E−04	1.48	0.247
Pathways in cancer	175	2.182	4.44E−04	1.21	0.554
Non-small cell lung cancer	37	0.461	0.001	1.55	0.789
**Insulin signaling pathway**	79	0.985	0.001	1.33	0.998
Chronic myeloid leukemia	48	0.599	0.001	1.45	1.035
Neurotrophin signaling pathway	73	0.91	0.001	1.34	1.294
Thyroid cancer	22	0.274	0.002	1.72	2.27
Glioma	40	0.499	0.003	1.44	3.824
**Graft-versus-host disease**	27	0.337	0.004	1.57	4.376
Melanogenesis	58	0.723	0.004	1.33	5.287
Allograft rejection	25	0.312	0.005	1.58	6.164
**Fatty acid metabolism**	27	0.337	0.006	1.53	7.216
Type I diabetes mellitus	28	0.349	0.006	1.51	7.697
Bladder cancer	28	0.349	0.006	1.51	7.697
Endocytosis	99	1.234	0.007	1.22	7.938
**Adherens junction**	46	0.574	0.007	1.36	8.848
Gap junction	52	0.648	0.008	1.33	9.194
Proteasome	30	0.374	0.011	1.45	12.807
Lysine degradation	28	0.349	0.015	1.44	17.473
Glycine, serine and threonine metabolism	21	0.262	0.017	1.54	19.492
Citrate cycle (TCA cycle)	21	0.262	0.017	1.54	19.492
Acute myeloid leukemia	35	0.436	0.018	1.37	19.852
p53 signaling pathway	40	0.499	0.018	1.33	20.5
Long-term potentiation	40	0.499	0.018	1.33	20.5
Pancreatic cancer	42	0.524	0.018	1.32	20.5
Drug metabolism	27	0.337	0.022	1.42	24.2
Glycerolipid metabolism	28	0.349	0.022	1.41	24.6
**Apoptosis**	49	0.611	0.023	1.28	25.4
**Adipocytokine signaling pathway**	39	0.486	0.024	1.32	26.5
Small cell lung cancer	47	0.586	0.03	1.27	31.9
Glycolysis/Gluconeogenesis	35	0.436	0.033	1.32	34
**VEGF signaling pathway**	42	0.524	0.041	1.27	40.5
**Wnt signaling pathway**	78	0.973	0.049	1.17	46.4
Colorectal cancer	46	0.574	0.049	1.24	46.5
Valine, leucine and isoleucine degradation	26	0.324	0.06	1.34	53.7
Pyruvate metabolism	24	0.299	0.06	1.36	53.8
Circadian rhythm	10	0.125	0.061	1.75	54.3
Calcium signaling pathway	89	1.11	0.062	1.15	55.1
Glyoxylate and dicarboxylate metabolism	11	0.137	0.066	1.66	57.6
GnRH signaling pathway	52	0.648	0.066	1.2	57.7
Lysosome	61	0.761	0.067	1.18	58.1
Viral myocarditis	39	0.486	0.067	1.25	58.3
**PPAR signaling pathway**	38	0.474	0.069	1.25	58.8
Long-term depression	38	0.474	0.069	1.25	58.8
Spliceosome	65	0.81	0.074	1.17	61.5
Pathogenic Escherichia coli infection	32	0.399	0.075	1.27	62
Arginine and proline metabolism	30	0.374	0.076	1.28	63
Autoimmune thyroid disease	29	0.362	0.077	1.29	63.4
Type II diabetes mellitus	27	0.337	0.079	1.3	64.2
Fatty acid elongation in mitochondria	7	0.087	0.08	1.99	64.6
Aldosterone-regulated sodium reabsorption	24	0.299	0.081	1.33	65.2
**Natural killer cell mediated cytotoxicity**	68	0.848	0.081	1.16	65.2
Asthma	18	0.224	0.082	1.41	65.5
Alzheimer’s disease	82	1.022	0.082	1.14	65.6
**T cell receptor signaling pathway**	56	0.698	0.088	1.18	68.3
**Hedgehog signaling pathway**	31	0.387	0.096	1.26	71.6
mTOR signaling pathway	29	0.362	0.099	1.27	72.8

The analysis was performed using the KEGG database. Pathways of putative relevance to the pathogenesis of chronic lymphedema are indicated in bold font. Additional pathway analysis with GeneSpring identified three additional significant pathways: transforming growth factor beta receptor (TGFBR); epidermal growth factor receptor 1 (EGFR1) and interleukin 6 (IL-6).

### High-Throughput Protein Assay and Logistic Regression Modeling

With our goal of determining potential protein targets that discriminate the presence of lymphatic vascular insufficiency when assayed in serum, we took the microarray analysis described above and directed our attention to the subset of identified, differentially expressed genes that encode secreted proteins, as delineated in the DAVID bioinformatic database. Seventy-four secreted proteins of interest were identified (Table S2), of which 42 were deemed potentially suitable for a potential high throughput serum assay (Table 3). For a high throughput method of testing proteins, the Luminex 51-plex Human Cytokines system (Affymetrix Panomic Solutions, Santa Clara, CA) was selected (Table 4), because a high number (*viz*., 15) of proteins in the assay system figured among our list of proteins of interest; furthermore, two supplemental proteins within the multiplex assay (basic FGF and TNF beta) were identified as plausible surrogates for an additional pair of our identified proteins-of-interest. In addition, the Luminex 51-plex features leptin; while the gene encoding leptin was not statistically upregulated in our discovery assay, we chose to include it in the serum protein analysis because if the central role of altered adipose biology in the natural history of human lymphedema [4].

**Table 3:** Secreted protein targets identified by paired microarray.

Gene	Fold Change	P value
Adrenomedullin	1.3	0.04
Alpha-1B glycoprotein	1.4	0.002
Angiopoietin 1	1.9	0.0003
Apolipoprotein A1	1.5	0.0002
Chemokine (c-C motif) ligand 25	1.5	0.0009
Chemokine (C-X-C) motif ligand 17	1.7	0.0003
Chorionic somatomammotropin hormone 1	2.1	2.4×10^−5^
Chromogranin A	2.3	1.2×10^−5^
Chymotrypsinogen B2	1.8	0.0001
Cytokine-like 1	1.5	8.3×10^−5^
Hepatocyte growth factor	1.6	0.002
Immunoglobulin kappa variable 4-1	2.1	0.0003
Interferon, alpha 2	1.6	0.002
Inteferon, alpha 21	1.6	0.0007
Interferon, alpha 4	1.6	0.0006
Interferon, alpha 5	1.4	0.003
Interferon, alpha 8	1.4	0.009
Interferon, gamma	1.6	0.002
Interleukin 10	1.9	0.0004
Interleukin 13	1.3	0.01
Interleukin 17A	1.6	0.0004
Interleukin 17F	1.8	0.001
Interleukin 19	1.4	0.001
Interleukin 20	1.8	7.7×10^−5^
Interleukin 24	1.5	7.2×10^−5^
Interleukin 25	1.6	0.01
Interleukin 26	1.6	0.002
Interleukin 28A	1.7	0.0009
Interleukin 4	1.8	9×10^−5^
Interleukin 6	1.6	0.002
Interferon, alpha 21	2.0	3.5×10^−7^
Kallikrein-related peptidase 3	2.3	1.9×10^−5^
Lipoprotein a	1.5	0.002
Pentraxin 3	2.4	1.8×10^−6^
Phospholipase A2	1.7	2×10^−6^
Plasminogen	1.5	0.004
Renin	1.8	0.0004
Serpin peptidase inhibitor, clade E, member 1 (PAI 1)	1.9	0.0002
Thrombopoietin	1.5	0.002
Transforming growth factor beta	1.4	0.02
Tumor necrosis factor	1.8	0.0006
Vascular endothelial growth factor A	1.5	0.0007

The genes listed were upregulated in the lymphedema specimens when compared to the paired normal tissues derived from the same subjects and encode secreted proteins of potential interest. The p-values are not corrected for multiple comparisons.

**Table 4:** The Luminex 51-plex assay.

Epithelial neutrophils-activating protein 78	Interleukin 17F
Eotaxin	***Leptin***
**Fibroblast growth factor (basic)** *	Leukemia inhibitory factor
Granulocyte colony stimulating factor	Macrophage inflammatory protein 1-alpha
Granulocyte macrophage stimulating factor	Macrophage inflammatory protein 1-beta
Growth regulated oncogene-alpha	Monocyte chemotactic protein-1
**Hepatocyte growth factor**	Monocyte chemotactic protein-3
**Interferon-alpha**	Macrophage colony-stimulating factor
Interferon-beta	Monokine-induced by Interferon- gamma
**Intereron-gamma**	Nerve growth factor
Interferon gamma-induced protein 10	**Plasminogen activator inhibitor-1**
**Interleukin 1- alpha**	Platelet-derived growth factor-BB
**Interleukin 1-beta**	RANTES
Interleukin 1 receptor antagonist	Resistin
Interleukin 2	Soluble CD40 Ligand
**Interleukin 4**	Stem cell factor
Interleukin 5	sFAS ligand
Interleukin 6	Soluble intercellular adhesion molecule-1
Interleukin 7	Soluble vascular cell adhesion molecule-1
Interleukin 8	Transforming growth factor-alpha
**Interleukin 10**	**Transforming growth factor-beta**
**Interleukin 12p40**	Tumor necrosis factor-alpha
**Interleukin 12p70**	**Tumor necrosis factor-beta** §
**Interleukin 13**	TNF-related apoptosis-inducing ligand
Interleukin 15	**Vascular endothelial growth factor**
**Interleukin 17A**	

For the purposes of this investigation, we limited our analysis to the 17 highlighted proteins, as identified in the prospective transcriptomic analysis; in addition, in order to discriminate the altered adipose biology of chronic lymphedema, we also assessed the differential expression of leptin in lymphedema sera *vs.* normals.

* surrogate for fibroblast growth factor 10.

§ surrogate for tumor necrosis superfamily ligand.

The Luminex assay was performed on serum collected from the original 27 lymphedema patients as well as from 12 healthy normal subjects: while the multiplex assay generated data for 51 targets, only the aforementioned set of 18 proteins (including leptin) was analyzed. A logistic regression model was fitted with these levels as features, to discriminate between the two groups of subjects. To pare down even further the number of proteins, an L1-regularization term was included in the cost function. The final model included 6 of the original 18 proteins (Table 5); interestingly, the regression model yielded candidates that are associated with the major biological characteristics of chronic lymphedema (lymphangiogenesis, inflammation, fibrosis, and adipocytokine signaling), as identified clinically and by the previously discussed pathway analysis.

**Table 5:** Logistic regression modeling of high throughput protein assay.

Protein	P-value
**Lymphangiogenesis**	
FGFb	0.03
**Inflammation**	
IL4	0.04
IL10	0.05
TNFb	0.3
**Fibrosis**	
TGFb	0.004
**Adipocytokine signaling**	
Leptin	1.8×10^−5^

Eighteen proteins were assayed by the Luminex bead assay, comparing the sera of 27 lymphedema subjects with 12 healthy controls. The p-values reflect the significance of differences between lymphedema subjects and normals. Of note is the fact that leptin is highly differentially detected in lymphedema *vs.* normal, despite the absence of differential expression by transcriptomic analysis. FGFb, basic fibroblast growth factor; IL4, interleukin 4; IL10, interleukin 10; TNFb, tumor necrosis factor beta; TGFb, transforming growth factor beta.

### Prospective Target Validation and Creation of the Receiver Operating Characteristic

**Figure 3:**
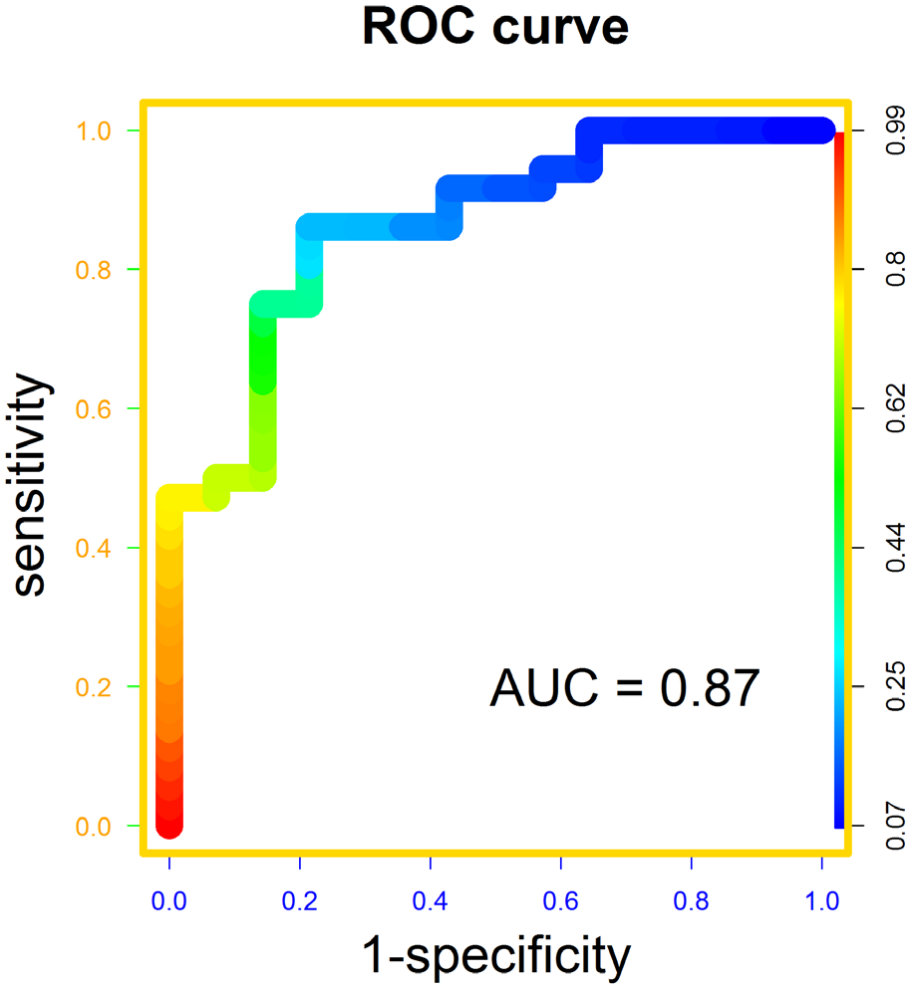
Receiver operating characteristic curve. Based on the L1-regularized logistic regression model with six proteins, a receiver operating characteristic (ROC) curve yields an area under the curve (AUC) of 0.87. The color at a position along the curve is indicative of the specificity, and sensitivity can be gauged by looking at the color at the corresponding height along the left, vertical axis.

Because a model fit on the training data is typically over-optimistic, we collected sera from an independent set of cases and controls for model validation. We performed the 51-plex Luminex assay on sera collected from 36 lymphedema subjects and 15 normal healthy adults. Our analysis focused solely on the 6 proteins determined to be of interest from the previous stage of biomarker identification. Using the L1-regularized logistic model, the AUC of the ROC curve was found to be 0.87 (95% CI: 0.75 to 0.97) (Figure 3). Five of the six proteins had significant p-values at the 0.05 level under the non-parametric Mann-Whitney U-test (Table 5) with all protein levels trending to higher values among the affected subjects.

## Discussion

Our specific approach to the identification of useful disease-related circulating biomarkers is derived from initial observations in a murine experimental model of acquired lymphedema [10]. The prior study demonstrated that, in the skin, lymphedema invokes altered expression of many biological processes, including the acute inflammatory response, wound healing and fibrosis, angiogenesis, cytoskeletal organization, *Wnt* pathway activation, and adipogenesis. Guided by these results, our goal in this investigation was to identify circulating proteins that are associated with the altered biology of target tissues in human lymphedema. We utilized a transcriptomic approach to the identification of potential candidates, using paired microarray analysis of skin biopsy specimens derived from normal and affected regions within the same patient (Figure 1). From the list of differentially expressed genes in this discovery step, we identified the smaller subset for which there is a secreted protein gene product. We then employed a multiplex protein assay to examine whether a subset of these identified proteins would be suitable, through serum assay, to prospectively distinguish lymphedema patients from normals. A fitted model purposefully constructed to induce sparsity was used to construct a final selected panel of six proteins and demonstrated the ability to discriminate diseased individuals from normals.

The study design employed in this work is distinctive. Much of the current, aggressive search for biomarkers in cancer and other disease has focused largely on a proteomic approach to identify these distinctive molecular signatures, but this approach has met with varying success [25], [26]. In the current investigation, we have chosen to utilize the somewhat less commonly applied, but effective [24], [27]–[33], transcriptomics-first approach to inform the proteomics.

The validation of a sensitive and specific biomarker assay for human acquired lymphatic vascular insufficiency represents a significant development for the disease community. The Executive Summary of the 2007 Trans-NIH Working Group on Lymphatics underscores the importance of biomarker identification for lymphatic diseases, designating this as one of its seven concrete recommendations [6]. An accurate bioassay for the disease state should help to pave the road for future human clinical trials of experimental drugs and therapies designed to treat human lymphatic diseases [34].

This biomarker panel will not supplant the utility of an accurate bedside evaluation of established lymphedema; however, it is likely that an accurate biomarker assay, once fully developed and validated, will be useful in risk stratification and in the early detection of latent and subtle disease. Markers of disease are likely to be important, since late activating events often precipitate progression from latent disease in humans. In lymphedema, this concept is supported by our earlier work in both murine and human disease Increased expression of endogenous danger signal proteins heralds progressive tissue pathology. These findings suggest that lymph stasis is an injury stimulus that initiates and perpetuates disease [35].

The utility of this biomarker panel for risk stratification must be prospectively evaluated. One of the elusive attributes of acquired lymphatic vascular insufficiency is its strong, often protracted, tendency toward clinical latency [36]. In other words, the anatomic insult is sustained by the patient at a point in time that may precede, by up to 5 years or longer, the clinically recognizable, functional expression of disease. Published studies of the incidence of breast cancer-associated lymphedema illustrate this phenomenon well [37]. Furthermore, If one assumes that 20% of breast cancer survivors are at clinical risk to develop lymphedema, how are these individuals to be distinguished from the remaining 80% that remain disease-free? Most of the surgical and other clinical variables have no statistically-identifiable bearing on relative risk [37]. The few treatment variables that are known to enhance risk do not, alone, accurately distinguish the sizeable at-risk subpopulation [38]. Taken together, these disease attributes significantly hamper risk-stratification and efforts at early therapeutic intervention for acquired lymphedema, despite the growing evidence that early therapeutic intervention has the capacity to significantly alter the course of the disease [39].

A further impetus to the development of sensitive and accurate diagnostic modalities resides in the growing likelihood that molecular and pharmacological approaches to therapy might soon be feasible [40]–[44]. Thus, there is a future need for appropriate clinical tools to detect latent disease and its responses to therapeutic intervention.

Our study harbors several potential limitations. Ideally, the most significant transcripts identified in the microarray experiments would have been carried forward to the protein assay, as they would have been anticipated to be most readily and differentially quantifiable in the disease-related sera. However, these identified candidates do not universally possess commercially available antibodies that are suitable for reduction into a quantifiable assay. For practicality, we chose our particular 51-plex Luminex assay as a compromise between our ability to study candidates of theoretical interest and the feasibility and cost of generating data on an extensive list of proteins to be studied within a large number of patient-derived sera. A corollary limitation may exist in our decision, based upon the transcriptomic discovery step, to limit our statistical analysis to a pre-chosen set of 18 out of the 51 proteins represented on the multiplex assay. This, in turn, yielded the final set of 6 protein biomarkers, for which data was generated through the two stages of model fitting and validation, respectively. Although it may be considered inefficient to discard such large portions of potentially meaningful data, the alternative approach did not ultimately seem efficient or economically suitable to our pragmatic endpoint.

Ultimately, the concluding high AUC that was derived from the validation set lends credence to the pragmatic strategy that was chosen. In addition, the biomarkers of this assay may serve as actual pharmaceutical targets. The observation that these genes are differentially expressed in diseased and normal tissues within the same individual, and that the secreted protein products of these genes are incrementally detected in lymphedema sera when compared to those of healthy controls, suggests that these proteins are directly related to the pathogenesis of the tissue pathology in lymphedema. This conclusion is buttressed by the observation that proteins representative of four of the central pathogenetic modalities–lymphangiogensis, inflammation, fibrosis, and altered lipid metabolism [4], [35], [45]–[49] –are contained in our final proposed multi-marker assay. Nevertheless, we cannot exclude the possibility that, had more individual studies been carried out, the list of proteins in our final model may have been different, at least in part.

In conclusion, we have identified and prospectively validated the ability of a newly identified circulating molecular signature to accurately discriminate established disease patients from a cohort of normal subjects. Further studies are warranted to determine whether this newly-identified biomarker panel will possess utility as an instrument for *in vitro* diagnosis of early and latent disease; the ultimate applicability to risk stratification, quantitation of disease burden, and response to therapy can easily be envisioned.

## Supporting Information

Table S1 **300 differentially-expressed genes with the lowest observed p-values.**
(DOCX)

Table S2 **Secreted proteins of interest identified by microarray analysis.**
(DOC)
