# Supplementary material for: Prospective Transcriptomic Pathway Analysis of Human Lymphatic Vascular Insufficiency: Identification and Validation of a Circulating Biomarker Panel
Source: PLoS One. 2012 Dec 18;7(12):e52021. doi: 10.1371/journal.pone.0052021 (PMC3525657; doi:10.1371/journal.pone.0052021)
Supplement: Table S1 — 300 differentially-expressed genes with the lowest observed p-values. (DOCX) [file pone.0052021.s001.docx]

**TABLE S1**

| SYMBOL | GENE DESCRIPTION | ACCESSION NUMBER | EXPRESSION RATIO | P VALUE |
| --- | --- | --- | --- | --- |
|  |  |  |  |  |
| MGC8386 | melanoma antigen family D, 2 | NM_201222 | -1.5 | 6.24E-11 |
| DKFZp434A132 | protocadherin 21 | NM_033100 | -1.3 | 8.56E-11 |
| DKFZp686N0152 | cathepsin B | NM_147780 | -1.2 | 2.49E-10 |
| ASS1 | argininosuccinate synthetase 1 | NM_054012 | -1.2 | 2.81E-10 |
| PC3 | ARP1 actin-related protein 1 homolog B, centractin beta (yeast) | NM_005735 | -1.2 | 3.47E-10 |
| dJ393P12.2 | WAS protein family, member 2 | NM_006990 | -1.3 | 3.99E-10 |
| FLJ31401 | dual specificity phosphatase 5 pseudogene | AK055963 | 1.6 | 4.22E-10 |
| CEBPA | CCAAT/enhancer binding protein (C/EBP), alpha | NM_004364 | -1.5 | 4.93E-10 |
| IDH | isocitrate dehydrogenase 2 (NADP+), mitochondrial | NM_002168 | -1.4 | 5.49E-10 |
| lip2 | annexin A2 pseudogene 3; annexin A2; annexin A2 pseudogene 1 | NR_001562 | -1.3 | 1.43E-09 |
| MAN2C1 | mannosidase, alpha, class 2C, member 1 | NM_006715 | -1.1 | 1.62E-09 |
| MIG10 | phosphoglycerate kinase 1 | NM_000291 | -1.1 | 1.84E-09 |
| F2RL3 | coagulation factor II (thrombin) receptor-like 3 | ENST00000248076 | 1.0 | 1.89E-09 |
| gapd | glyceraldehyde-3-phosphate dehydrogenase-like 6; hypothetical protein LOC100133042; glyceraldehyde-3-phosphate dehydrogenase | NM_002046 | -1.1 | 2.02E-09 |
| SNX17 | sorting nexin 17 | NM_014748 | -1.3 | 2.03E-09 |
| HLA-CDA12 | major histocompatibility complex, class I, J (pseudogene) | ENST00000376793 | -1.3 | 2.91E-09 |
| LBH | chromosome 6 open reading frame 1 | NM_178508 | -1.1 | 2.91E-09 |
| HLA-H | major histocompatibility complex, class I, H (pseudogene) | NR_001434 | -1.3 | 3.25E-09 |
| MGC131998 | mitochondrial carrier homolog 1 (C. elegans) | NM_014341 | -1.0 | 4.19E-09 |
| xp33 | chromosome 1 open reading frame 46 | ENST00000323057 | -1.3 | 5.32E-09 |
| 40457 | POU class 3 homeobox 1 | NM_002699 | -1.4 | 5.66E-09 |
| ECH1 | enoyl Coenzyme A hydratase 1, peroxisomal | NM_001398 | -1.1 | 6.02E-09 |
| Kepi | protein phosphatase 1, regulatory (inhibitor) subunit 14C | NM_030949 | -1.2 | 6.06E-09 |
| GNK | N-acetylglucosamine kinase | NM_017567 | -1.0 | 6.55E-09 |
| dJ211D12.5 | WAP four-disulfide core domain 5 | NM_145652 | -1.2 | 8.45E-09 |
| Mdu1 | solute carrier family 3 (activators of dibasic and neutral amino acid transport), member 2 | NM_002394 | -1.2 | 9.53E-09 |
| VMA2 | ATPase, H+ transporting, lysosomal 56/58kDa, V1 subunit B2 | NM_001693 | -0.8 | 9.83E-09 |
| GLTSCR2 | glioma tumor suppressor candidate region gene 2; glioma tumor suppressor candidate region gene 2 pseudogene | NM_015710 | -1.3 | 1.02E-08 |
| RUTBC3 | small G protein signaling modulator 3 | NM_015705 | -1.0 | 1.02E-08 |
| mixl | Mix1 homeobox-like 1 (Xenopus laevis) | NM_031944 | 1.3 | 1.06E-08 |
| FLJ20296 | retinol saturase (all-trans-retinol 13,14-reductase) | NM_017750 | -1.2 | 1.07E-08 |
| PDI | prolyl 4-hydroxylase, beta polypeptide | NM_000918 | -1.0 | 1.14E-08 |
| GPD1 | glycerol-3-phosphate dehydrogenase 1 (soluble) | NM_005276 | -1.6 | 1.16E-08 |
| Hnf3g | forkhead box A3 | NM_004497 | 1.6 | 1.34E-08 |
| PSORS1 | major histocompatibility complex, class I, C; major histocompatibility complex, class I, B | BC091497 | -1.2 | 1.54E-08 |
| PRAD1 | cyclin D1 | NM_053056 | -1.2 | 1.69E-08 |
| manB | mannosidase, alpha, class 2B, member 1 | NM_000528 | -1.0 | 1.71E-08 |
| LOC644063 | heterogeneous nuclear ribonucleoprotein K; similar to heterogeneous nuclear ribonucleoprotein K | XR_018217 | -1.2 | 1.81E-08 |
| Ubc-rs2 | ubiquitin-conjugating enzyme E2M (UBC12 homolog, yeast); ubiquitin-conjugating enzyme E2M pseudogene 1 | NM_003969 | -1.3 | 1.86E-08 |
| 40462 | POU class 2 homeobox 3 | NM_014352 | -1.0 | 1.90E-08 |
| MGC10763 | interleukin 17 receptor C | NM_153461 | -1.0 | 1.92E-08 |
| Ng23 | chromosome 6 open reading frame 26 | NM_001039651 | -0.9 | 2.03E-08 |
| PLEKHM2 | pleckstrin homology domain containing, family M (with RUN domain) member 2 | BC068599 | -1.1 | 2.06E-08 |
| gapd | glyceraldehyde-3-phosphate dehydrogenase-like 6; hypothetical protein LOC100133042; glyceraldehyde-3-phosphate dehydrogenase | NM_002046 | -1.2 | 2.11E-08 |
| FLJ10066 | activating transcription factor 6 beta | NM_004381 | 1.7 | 2.22E-08 |
| PSORS1 | major histocompatibility complex, class I, C; major histocompatibility complex, class I, B | BC091497 | -1.1 | 2.27E-08 |
| pef1 | penta-EF-hand domain containing 1 | NM_012392 | -1.1 | 2.33E-08 |
| PSORS1 | major histocompatibility complex, class I, C; major histocompatibility complex, class I, B | BC091497 | -1.0 | 2.41E-08 |
| 3PGDH | phosphoglycerate dehydrogenase | NM_006623 | -1.0 | 2.41E-08 |
| MGC17525 | solute carrier family 25 (mitochondrial carrier; adenine nucleotide translocator), member 6 | NM_001636 | -1.1 | 2.82E-08 |
| Psmc4 | similar to 26S protease regulatory subunit 6B (MIP224) (MB67-interacting protein) (TAT-binding protein 7) (TBP-7); proteasome (prosome, macropain) 26S subunit, ATPase, 4 | NM_006503 | -0.9 | 2.88E-08 |
| MGC131997 | valosin-containing protein | NM_007126 | -1.4 | 3.01E-08 |
| SREBF1 | sterol regulatory element binding transcription factor 1 | NM_001005291 | -1.0 | 3.05E-08 |
| gapd | glyceraldehyde-3-phosphate dehydrogenase-like 6; hypothetical protein LOC100133042; glyceraldehyde-3-phosphate dehydrogenase | NM_002046 | -1.1 | 3.15E-08 |
| FLJ12389 | acetoacetyl-CoA synthetase | NM_023928 | -1.0 | 3.15E-08 |
| PBR | translocator protein (18kDa) | NM_007311 | -1.1 | 3.17E-08 |
| ALDHI | aldehyde dehydrogenase 2 family (mitochondrial) | NM_000690 | -0.7 | 3.22E-08 |
| SWP1 | ribophorin II | NM_002951 | -0.9 | 3.28E-08 |
| h37 | RNA binding motif protein 5 | NM_005778 | -1.2 | 3.34E-08 |
| AFAR2 | aldo-keto reductase family 7, member A3 (aflatoxin aldehyde reductase) | NM_012067 | -1.1 | 3.37E-08 |
| rpb5 | polymerase (RNA) II (DNA directed) polypeptide E, 25kDa | NM_002695 | -1.1 | 3.42E-08 |
| MGC71256 | hypothetical LOC152217 | BC062368 | -1.0 | 3.47E-08 |
| C22orf9 | chromosome 22 open reading frame 9 | NM_015264 | -1.0 | 3.55E-08 |
| HSD28 | HRAS-like suppressor | NM_020386 | -0.9 | 3.63E-08 |
| MANSC2 | serine peptidase inhibitor, Kunitz type 1 | NM_003710 | -1.2 | 3.63E-08 |
| PSORS1 | major histocompatibility complex, class I, C; major histocompatibility complex, class I, B | BC091497 | -1.0 | 3.71E-08 |
| MGC102966 | similar to Keratin, type I cytoskeletal 16 (Cytokeratin-16) (CK-16) (Keratin-16) (K16) | BC110641 | -1.5 | 3.82E-08 |
| Rab2l | ral guanine nucleotide dissociation stimulator-like 2 | NM_004761 | -1.0 | 3.84E-08 |
| gapd | glyceraldehyde-3-phosphate dehydrogenase-like 6; hypothetical protein LOC100133042; glyceraldehyde-3-phosphate dehydrogenase | NM_002046 | -1.0 | 3.95E-08 |
| MGC29814 | family with sequence similarity 100, member B | NM_182565 | -0.8 | 3.99E-08 |
| BCDase | N-acylsphingosine amidohydrolase (non-lysosomal ceramidase) 2C; N-acylsphingosine amidohydrolase (non-lysosomal ceramidase) 2 | NM_019893 | 1.5 | 4.02E-08 |
| b3gat3 | beta-1,3-glucuronyltransferase 3 (glucuronosyltransferase I) | NM_012200 | -1.1 | 4.06E-08 |
| ANPa | natriuretic peptide receptor A/guanylate cyclase A (atrionatriuretic peptide receptor A) | NM_000906 | -0.8 | 4.17E-08 |
| CAP1-PEN | CAP, adenylate cyclase-associated protein 1 (yeast) | NM_006367 | -1.2 | 4.31E-08 |
| 40239 | membrane-associated ring finger (C3HC4) 2 | NM_016496 | -1.0 | 4.47E-08 |
| PRAD1 | cyclin D1 | NM_053056 | -1.1 | 4.48E-08 |
| Ptgs1 | prostaglandin-endoperoxide synthase 1 (prostaglandin G/H synthase and cyclooxygenase) | NM_000962 | -1.0 | 4.90E-08 |
| Celf3 | trinucleotide repeat containing 4 | NM_007185 | 1.2 | 4.91E-08 |
| APEH | N-acylaminoacyl-peptide hydrolase | NM_001640 | -1.0 | 5.04E-08 |
| Fam108a1 | family with sequence similarity 108, member A4; family with sequence similarity 108, member A5; family with sequence similarity 108, member A1 | NM_031213 | -1.0 | 5.09E-08 |
| PP1201 | transmembrane BAX inhibitor motif containing 1 | NM_022152 | -1.2 | 5.30E-08 |
| bscL | 1-acylglycerol-3-phosphate O-acyltransferase 2 (lysophosphatidic acid acyltransferase, beta) | NM_006412 | -1.6 | 5.33E-08 |
| Npm2 | nucleophosmin/nucleoplasmin, 2 | NM_182795 | 1.6 | 5.83E-08 |
| FLJ45062 | protein tyrosine phosphatase, receptor type, F | NM_002840 | -1.2 | 5.92E-08 |
| CICE | cell death-inducing DFFA-like effector c pseudogene | NR_002786 | -1.5 | 5.99E-08 |
| Ubc-rs2 | ubiquitin-conjugating enzyme E2M (UBC12 homolog, yeast); ubiquitin-conjugating enzyme E2M pseudogene 1 | NR_002837 | -1.1 | 6.07E-08 |
| RHOHP1 | ras homolog gene family, member D | NM_014578 | -1.3 | 6.56E-08 |
| RPS2_14_794 | ribosomal protein S2 pseudogene 32 | BC026177 | -1.0 | 6.75E-08 |
| Ol-64 | serpin peptidase inhibitor, clade A (alpha-1 antiproteinase, antitrypsin), member 12 | NM_173850 | -1.2 | 7.17E-08 |
| DKFZp434M0519 | transmembrane protein 55B | NM_144568 | -1.1 | 7.27E-08 |
| ARF5 | ADP-ribosylation factor 5 | NM_001662 | -1.1 | 7.31E-08 |
| CPSF3L | cleavage and polyadenylation specific factor 3-like | NM_017871 | -1.0 | 7.32E-08 |
| Eif2b5 | eukaryotic translation initiation factor 2B, subunit 5 epsilon, 82kDa | NM_003907 | -1.0 | 7.34E-08 |
| Iglon5 | IgLON family member 5 | ENST00000270642 | 1.5 | 7.45E-08 |
| Tspan28 | CD81 molecule | NM_004356 | -0.9 | 7.49E-08 |
| GRASP-1 | GRIP1 associated protein 1 | NM_020137 | -1.1 | 7.57E-08 |
| LOC283587 | hypothetical protein LOC283587 | BC029479 | 1.1 | 7.71E-08 |
| FLJ10901 | chromosome 1 open reading frame 106 | NM_018265 | -1.0 | 7.80E-08 |
| A2RP | ataxin 2-like | NM_145714 | -0.7 | 7.92E-08 |
| MK2 | mitogen-activated protein kinase-activated protein kinase 2 | NM_004759 | -1.1 | 8.05E-08 |
| Wrch2 | ras homolog gene family, member V | NM_133639 | -1.2 | 8.05E-08 |
| MGC126031 | histone cluster 2, H3, pseudogene 2 | ENST00000369176 | -0.8 | 8.27E-08 |
| RAF1 | v-raf-1 murine leukemia viral oncogene homolog 1 | NM_002880 | -0.8 | 8.45E-08 |
| MMIb | myosin IC | NM_033375 | -1.2 | 8.55E-08 |
| ekv | gap junction protein, beta 3, 31kDa | NM_024009 | -0.9 | 8.79E-08 |
| DKFZp762M136 | fem-1 homolog a (C. elegans); similar to fem-1 homolog a (C.elegans); similar to fem-1 homolog a | NM_018708 | -1.1 | 9.00E-08 |
| OTB1 | OTU domain, ubiquitin aldehyde binding 1 | NM_017670 | -1.1 | 9.14E-08 |
| RAF1 | v-raf-1 murine leukemia viral oncogene homolog 1 | NM_002880 | -0.9 | 9.15E-08 |
| ACAD-9 | acyl-Coenzyme A dehydrogenase family, member 9 | NM_014049 | -1.1 | 9.16E-08 |
| FLJ45200 | AFG3 ATPase family gene 3-like 1 (S. cerevisiae) | NR_003226 | -1.0 | 9.19E-08 |
| DKFZp434M202 | tudor domain containing 10 | NM_182499 | 2.0 | 9.30E-08 |
| MGC15959 | H1 histone family, member X | NM_006026 | -1.2 | 9.46E-08 |
| IPP1 | protein phosphatase 1, regulatory (inhibitor) subunit 1A | NM_006741 | -1.6 | 9.86E-08 |
| MGC11268 | pseudouridylate synthase 1 | NM_025215 | -0.9 | 1.00E-07 |
| PSORS1 | major histocompatibility complex, class I, C; major histocompatibility complex, class I, B | BC091497 | -1.1 | 1.03E-07 |
| SCRN2 | secernin 2 | NM_138355 | -1.1 | 1.04E-07 |
| FDPSL2A | MGC44478 | NR_003262 | -1.0 | 1.05E-07 |
| Hox-2.7 | homeobox B3 | NM_002146 | 0.7 | 1.05E-07 |
| LEP14 | late cornified envelope 3B | NM_178433 | -1.4 | 1.06E-07 |
| SLC25A4 | solute carrier family 25 (mitochondrial carrier; adenine nucleotide translocator), member 4 | NM_001151 | -1.1 | 1.07E-07 |
| MGC16491 | family with sequence similarity 46, member B | NM_052943 | -1.0 | 1.10E-07 |
| RPN9 | proteasome (prosome, macropain) 26S subunit, non-ATPase, 13 | NM_175932 | -1.3 | 1.10E-07 |
| TWF2 | twinfilin, actin-binding protein, homolog 2 (Drosophila) | NM_007284 | -1.1 | 1.11E-07 |
| tuba6 | tubulin, alpha 1c | NM_032704 | -0.8 | 1.14E-07 |
| RPL7AP30 | ribosomal protein L7a pseudogene 70; ribosomal protein L7a; ribosomal protein L7a pseudogene 30; ribosomal protein L7a pseudogene 66; ribosomal protein L7a pseudogene 27; ribosomal protein L7a pseudogene 11; ribosomal protein L7a pseudogene 62 | NM_000972 | -0.8 | 1.18E-07 |
| PSORS1 | major histocompatibility complex, class I, C; major histocompatibility complex, class I, B | BC091497 | -1.2 | 1.19E-07 |
| NTRK4 | discoidin domain receptor tyrosine kinase 1 | NM_013994 | -1.2 | 1.24E-07 |
| HHR23A | RAD23 homolog A (S. cerevisiae) | NM_005053 | -1.0 | 1.27E-07 |
| FLJ54370 | heat shock 70kDa protein 1A; heat shock 70kDa protein 1B | NM_005345 | -1.3 | 1.29E-07 |
| PRAD1 | cyclin D1 | NM_053056 | -1.2 | 1.35E-07 |
| FLJ38078 | glypican 1 | NM_002081 | -1.0 | 1.48E-07 |
| Cx31.1 | gap junction protein, beta 5, 31.1kDa | NM_005268 | -1.2 | 1.50E-07 |
| AGRNR | dystroglycan 1 (dystrophin-associated glycoprotein 1) | NM_004393 | -1.3 | 1.56E-07 |
| PSORS1 | major histocompatibility complex, class I, C; major histocompatibility complex, class I, B | BC091497 | -1.2 | 1.56E-07 |
| MRIP-1 | ArfGAP with GTPase domain, ankyrin repeat and PH domain 3 | NM_031946 | -1.3 | 1.56E-07 |
| RP1-28O10.2 | G0/G1switch 2 | NM_015714 | -1.8 | 1.60E-07 |
| FLJ11798 | F-box protein 17 | NM_024907 | -0.9 | 1.61E-07 |
| TGFBR3 | transforming growth factor, beta receptor III | NM_003243 | -1.0 | 1.65E-07 |
| MGC33315 | COMM domain containing 7 | NM_053041 | -1.0 | 1.67E-07 |
| RIGUI | period homolog 1 (Drosophila) | NM_002616 | -1.2 | 1.67E-07 |
| HSST2 | sulfotransferase family, cytosolic, 2B, member 1 | NM_004605 | -1.1 | 1.68E-07 |
| FLJ44681 | neurocan | NM_004386 | 1.3 | 1.69E-07 |
| LOC643446 | similar to ribonucleic acid binding protein S1; RNA binding protein S1, serine-rich domain | NM_006711 | -1.3 | 1.71E-07 |
| Stoml2 | stomatin (EPB72)-like 2 | NM_013442 | -0.9 | 1.75E-07 |
| DKFZp313H0740 | retinol dehydrogenase 13 (all-trans/9-cis) | NM_138412 | -1.1 | 1.80E-07 |
| MGC22217 | syndecan 4 | NM_002999 | -1.3 | 1.83E-07 |
| DYNII | dynamin 2 | NM_001005360 | -1.4 | 1.83E-07 |
| RNF221 | PHD and ring finger domains 1 | NM_020901 | -1.0 | 1.88E-07 |
| EIF4B | similar to eukaryotic translation initiation factor 4H; eukaryotic translation initiation factor 4B | NM_001417 | -1.0 | 1.97E-07 |
| cpeR | claudin 4 | NM_001305 | -1.3 | 2.03E-07 |
| FLJ30169 | tubulin, alpha 4a | NM_006000 | -1.1 | 2.04E-07 |
| pig5 | anoctamin 9 | NM_001012302 | -1.1 | 2.06E-07 |
| FLJ00082 | neurobeachin-like 2 | AK131104 | -1.0 | 2.07E-07 |
| FLJ40100 | tubulin, beta 8 | NM_177987 | -1.0 | 2.08E-07 |
| PRAD1 | cyclin D1 | NM_053056 | -1.0 | 2.09E-07 |
| zmat5 | zinc finger, matrin type 5 | NM_019103 | -0.9 | 2.12E-07 |
| MOB2 | HCCA2 protein | NM_053005 | -0.9 | 2.12E-07 |
| MTCH2 | mitochondrial carrier homolog 2 (C. elegans) | NM_014342 | -1.0 | 2.14E-07 |
| FLJ32791 | Mov10, Moloney leukemia virus 10, homolog (mouse) | NM_020963 | -1.2 | 2.14E-07 |
| ST-2 | syndecan binding protein (syntenin) 2 | NM_080489 | -1.0 | 2.14E-07 |
| TUBGCP6 | tubulin, gamma complex associated protein 6 | NM_020461 | -1.0 | 2.16E-07 |
| C/EBP-beta | CCAAT/enhancer binding protein (C/EBP), beta | NM_005194 | -0.9 | 2.20E-07 |
| SLC22A7 | solute carrier family 22 (organic anion transporter), member 7 | NM_153320 | 0.8 | 2.25E-07 |
| C1orf170 | chromosome 1 open reading frame 170 | AK123855 | 1.2 | 2.26E-07 |
| HASPP28 | PDGFA associated protein 1; similar to PDGFA associated protein 1 | NM_014891 | -1.0 | 2.28E-07 |
| QR2 | NAD(P)H dehydrogenase, quinone 2 | NM_000904 | -1.1 | 2.32E-07 |
| YY1 | YY1 transcription factor | NM_003403 | -0.9 | 2.33E-07 |
| PTP4A3 | protein tyrosine phosphatase type IVA, member 3 | NM_032611 | 1.4 | 2.36E-07 |
| PSORS1 | major histocompatibility complex, class I, C; major histocompatibility complex, class I, B | NM_005514 | -1.2 | 2.36E-07 |
| bHLHd14 | MLX interacting protein-like | NM_032951 | -1.3 | 2.41E-07 |
| efp | tripartite motif-containing 25 | NM_005082 | -1.3 | 2.43E-07 |
| FLJ20909 | enoyl Coenzyme A hydratase domain containing 3 | NM_024693 | -1.0 | 2.46E-07 |
| PRAD1 | cyclin D1 | NM_053056 | -1.4 | 2.51E-07 |
| FLJ35541 | nuclear receptor binding protein 1 | NM_013392 | -1.0 | 2.56E-07 |
| ezf | Kruppel-like factor 4 (gut) | NM_004235 | -1.0 | 2.60E-07 |
| NGRN | neugrin, neurite outgrowth associated | NM_016645 | -0.8 | 2.61E-07 |
| TP53TG3 | similar to TP53 target 3; TP53 target 3; similar to TP53TG3 protein | NM_016212 | 1.4 | 2.62E-07 |
| BCKDK | branched chain ketoacid dehydrogenase kinase | NM_005881 | -0.9 | 2.67E-07 |
| AP1B1 | adaptor-related protein complex 1, beta 1 subunit | NM_001127 | -1.1 | 2.72E-07 |
| TARBP-B | ribosomal protein L3; similar to 60S ribosomal protein L3 (L4) | NM_000967 | -0.8 | 2.78E-07 |
| ARSA-I | arsA arsenite transporter, ATP-binding, homolog 1 (bacterial) | NM_004317 | -0.8 | 2.82E-07 |
| CLSP | calmodulin-like 5 | NM_017422 | -1.1 | 2.83E-07 |
| Tmem45a | transmembrane protein 45A | NM_018004 | -1.3 | 2.84E-07 |
| DKFZp761G1923 | phosphatidylinositol 4-kinase type 2 alpha | NM_018425 | -1.0 | 2.85E-07 |
| TMEM179B | transmembrane protein 179B | NM_199337 | -0.9 | 2.87E-07 |
| dlx1 | distal-less homeobox 1 | NM_178120 | 1.3 | 2.92E-07 |
| JUP | junction plakoglobin | NM_002230 | -0.9 | 3.01E-07 |
| FLJ13705 | coiled-coil domain containing 69 | NM_015621 | 1.1 | 3.02E-07 |
| P/cl.18 | cleavage and polyadenylation specific factor 1, 160kDa | NM_013291 | -0.9 | 3.04E-07 |
| LOC100272216 | hypothetical LOC100272216 | AK130705 | -1.0 | 3.07E-07 |
| INPPL1 | inositol polyphosphate phosphatase-like 1 | NM_001567 | -0.9 | 3.13E-07 |
| MGC117217 | RAB5C, member RAS oncogene family | NM_201434 | -0.9 | 3.18E-07 |
| FBXW5 | F-box and WD repeat domain containing 5 | NM_018998 | -0.9 | 3.19E-07 |
| MGC119329 | RAR-related orphan receptor A | NM_134260 | -1.3 | 3.23E-07 |
| zirtl | solute carrier family 39 (zinc transporter), member 1 | NM_014437 | -1.0 | 3.31E-07 |
| MGC110993 | prosaposin | NM_002778 | -0.8 | 3.33E-07 |
| siat4 | ST3 beta-galactoside alpha-2,3-sialyltransferase 4 | NM_006278 | -1.0 | 3.36E-07 |
| gapd | glyceraldehyde-3-phosphate dehydrogenase-like 6; hypothetical protein LOC100133042; glyceraldehyde-3-phosphate dehydrogenase | NM_002046 | -0.9 | 3.36E-07 |
| BTBD6 | BTB (POZ) domain containing 6 | NM_033271 | -0.7 | 3.39E-07 |
| RAB11C | RAB25, member RAS oncogene family | NM_020387 | -1.1 | 3.40E-07 |
| IL26 | interleukin 26 | NM_018402 | 1.0 | 3.47E-07 |
| PRAD1 | cyclin D1 | NM_053056 | -1.2 | 3.49E-07 |
| GLTPP1 | glycolipid transfer protein; glycolipid transfer protein pseudogene 1 | NM_016433 | -1.2 | 3.56E-07 |
| Acdc | adiponectin, C1Q and collagen domain containing | NM_004797 | -1.3 | 3.65E-07 |
| npd | sphingomyelin phosphodiesterase 1, acid lysosomal | NM_000543 | -0.8 | 3.67E-07 |
| DKFZp686H1668 | StAR-related lipid transfer (START) domain containing 8 | NM_014725 | 1.3 | 3.72E-07 |
| KNS2 | kinesin light chain 1 | NM_182923 | -0.7 | 3.74E-07 |
| sctE | kallikrein-related peptidase 5 | NM_012427 | -1.1 | 3.75E-07 |
| DOK4 | docking protein 4 | NM_018110 | -0.9 | 3.77E-07 |
| fam43a | family with sequence similarity 43, member A | NM_153690 | -1.0 | 3.78E-07 |
| MGC24381 | chromosome 16 open reading frame 42 | NM_001001410 | -1.2 | 3.78E-07 |
| FLJ14425 | nischarin | NM_007184 | -1.1 | 3.79E-07 |
| SAND2 | MON1 homolog B (yeast) | NM_014940 | 1.5 | 3.79E-07 |
| MGC193256 | similar to WAS protein family homolog 1 | NM_182905 | -0.8 | 3.80E-07 |
| TARBP-B | ribosomal protein L3; similar to 60S ribosomal protein L3 (L4) | NM_000967 | -0.8 | 3.91E-07 |
| par-1 | MAP/microtubule affinity-regulating kinase 2 | NM_017490 | -1.2 | 4.11E-07 |
| CHPF | chondroitin polymerizing factor | NM_024536 | 1.4 | 4.16E-07 |
| mof | MYST histone acetyltransferase 1 | NM_032188 | -0.9 | 4.24E-07 |
| gapd | glyceraldehyde-3-phosphate dehydrogenase-like 6; hypothetical protein LOC100133042; glyceraldehyde-3-phosphate dehydrogenase | NM_002046 | -1.2 | 4.25E-07 |
| DKFZp779A1753 | microtubule-associated protein 4 | NM_002375 | -0.9 | 4.26E-07 |
| MAGED1 | melanoma antigen family D, 1 | NM_001005333 | -0.8 | 4.26E-07 |
| CGI69 | solute carrier family 25, member 39 | NM_016016 | -0.9 | 4.32E-07 |
| dJ794I6.3 | inosine triphosphatase (nucleoside triphosphate pyrophosphatase) | NM_033453 | -0.8 | 4.32E-07 |
| FAM108A6 | family with sequence similarity 108, member A6 | ENST00000333131 | -1.0 | 4.36E-07 |
| HLA-6.2 | major histocompatibility complex, class I, E | NM_005516 | -0.9 | 4.53E-07 |
| FLJ60124 | mitochondrial ribosomal protein L12 | NM_002949 | -1.1 | 4.54E-07 |
| rrp17 | RAS-like, family 10, member B | NM_033315 | 1.0 | 4.58E-07 |
| C2orf20 | kinesin family member 1A | NM_004321 | 1.0 | 4.58E-07 |
| nsep-1 | Y box binding protein 1 | NM_004559 | -0.9 | 4.79E-07 |
| PKBB | v-akt murine thymoma viral oncogene homolog 2 | NM_001626 | -0.9 | 4.81E-07 |
| *DHTR* | androgen receptor | NM_000044 | -0.9 | 4.86E-07 |
| Nop56 | NOP56 ribonucleoprotein homolog (yeast) | NM_006392 | -0.9 | 4.89E-07 |
| APOLV | apolipoprotein L, 5 | NM_030642 | 0.9 | 4.96E-07 |
| gapd | glyceraldehyde-3-phosphate dehydrogenase-like 6; hypothetical protein LOC100133042; glyceraldehyde-3-phosphate dehydrogenase | NM_002046 | -1.3 | 4.99E-07 |
| palm2 | A kinase (PRKA) anchor protein 2; paralemmin 2; PALM2-AKAP2 readthrough transcript | NM_147150 | 1.0 | 5.01E-07 |
| ADAMTSL5 | ADAMTS-like 5 | NM_213604 | 1.3 | 5.07E-07 |
| FLJ33325 | Thy-1 cell surface antigen | NM_006288 | 0.9 | 5.15E-07 |
| MGC903 | isocitrate dehydrogenase 3 (NAD+) beta | NM_174855 | -0.7 | 5.15E-07 |
| DYT5b | tyrosine hydroxylase | NM_199293 | 1.2 | 5.18E-07 |
| CXorf40B | chromosome X open reading frame 40A; chromosome X open reading frame 40B | NM_001013845 | -1.1 | 5.30E-07 |
| PRO2389 | WD repeat domain 23 | NM_025230 | -0.9 | 5.31E-07 |
| CFL | cofilin 1 (non-muscle) | NM_005507 | -1.1 | 5.37E-07 |
| TARBP-B | ribosomal protein L3; similar to 60S ribosomal protein L3 (L4) | NM_000967 | -1.0 | 5.38E-07 |
| Hebp2 | heme binding protein 2 | NM_014320 | -0.8 | 5.42E-07 |
| gapd | glyceraldehyde-3-phosphate dehydrogenase-like 6; hypothetical protein LOC100133042; glyceraldehyde-3-phosphate dehydrogenase | NM_002046 | -1.1 | 5.47E-07 |
| CEBPE | CCAAT/enhancer binding protein (C/EBP), epsilon | NM_001805 | -1.1 | 5.56E-07 |
| gapd | glyceraldehyde-3-phosphate dehydrogenase-like 6; hypothetical protein LOC100133042; glyceraldehyde-3-phosphate dehydrogenase | NM_002046 | -1.0 | 5.57E-07 |
| Muc5b | mucin 5B, oligomeric mucus/gel-forming | ENST00000349637 | 1.6 | 5.64E-07 |
| GIG35 | eukaryotic translation elongation factor 1 gamma | NM_001404 | -0.7 | 5.67E-07 |
| PSORS1 | major histocompatibility complex, class I, C; major histocompatibility complex, class I, B | BC091497 | -0.8 | 5.72E-07 |
| MGC4083 | tubulin, beta 6 | NM_032525 | -0.8 | 5.74E-07 |
| tuba6 | tubulin, alpha 1c | NM_032704 | -0.9 | 5.78E-07 |
| ppcA | cathepsin A | NM_000308 | -0.9 | 5.92E-07 |
| PLIN | perilipin | NM_002666 | -1.4 | 6.06E-07 |
| gapd | glyceraldehyde-3-phosphate dehydrogenase-like 6; hypothetical protein LOC100133042; glyceraldehyde-3-phosphate dehydrogenase | NM_002046 | -1.0 | 6.06E-07 |
| VGL | high density lipoprotein binding protein | AF116718 | 1.1 | 6.14E-07 |
| PH2 | glyoxylate reductase/hydroxypyruvate reductase | NM_012203 | -0.8 | 6.15E-07 |
| DESNUTRIN | patatin-like phospholipase domain containing 2 | NM_020376 | -0.9 | 6.17E-07 |
| MGC1126 | acyl-CoA thioesterase 7 | NM_181864 | -1.2 | 6.23E-07 |
| FLJ23436 | zinc finger protein 768 | NM_024671 | -0.7 | 6.66E-07 |
| PTE2 | acyl-CoA thioesterase 8 | NM_005469 | -0.7 | 6.68E-07 |
| FLJ12442 | 5'-nucleotidase domain containing 2 | NM_022908 | -1.0 | 6.78E-07 |
| Cdk11b | similar to cell division cycle 2-like 1 (PITSLRE proteins); cell division cycle 2-like 1 (PITSLRE proteins); cell division cycle 2-like 2 (PITSLRE proteins) | NM_033489 | -0.8 | 6.89E-07 |
| MGC87963 | glutathione S-transferase theta 2B (gene/pseudogene); glutathione S-transferase theta 2 | NM_000854 | -0.8 | 6.91E-07 |
| MEGF9 | multiple EGF-like-domains 9 | NM_001080497 | -0.9 | 6.99E-07 |
| SEMA6A | sema domain, transmembrane domain (TM), and cytoplasmic domain, (semaphorin) 6A | NM_020796 | 0.8 | 7.05E-07 |
| RNF108 | vacuolar protein sorting 11 homolog (S. cerevisiae) | NM_021729 | -0.8 | 7.11E-07 |
| H3.3A | H3 histone, family 3B (H3.3B); H3 histone, family 3A pseudogene; H3 histone, family 3A; similar to H3 histone, family 3B; similar to histone H3.3B | NM_005324 | -0.8 | 7.12E-07 |
| GPR56 | G protein-coupled receptor 56 | NM_201525 | -1.1 | 7.14E-07 |
| KIAA0773 | family with sequence similarity 131, member B | NM_014690 | 1.2 | 7.16E-07 |
| HARSL | histidyl-tRNA synthetase 2, mitochondrial (putative); D-tyrosyl-tRNA deacylase 1 homolog (S. cerevisiae) | NM_012208 | -1.0 | 7.23E-07 |
| GNIP | tripartite motif-containing 7 | NM_033342 | -1.1 | 7.38E-07 |
| HSF-27 | CDK5 regulatory subunit associated protein 3 | NM_176096 | -0.8 | 7.42E-07 |
| DKFZp762B1216 | mediator complex subunit 15 | NM_001003891 | -1.1 | 7.48E-07 |
| FLJ18582 | isochorismatase domain containing 2 | NM_024710 | -1.0 | 7.49E-07 |
| OR2M2Q | olfactory receptor, family 2, subfamily M, member 2 | NM_001004688 | 0.9 | 7.65E-07 |
| XPB | excision repair cross-complementing rodent repair deficiency, complementation group 3 (xeroderma pigmentosum group B complementing) | NM_000122 | -0.9 | 7.65E-07 |
| MKP4 | dual specificity phosphatase 9 | NM_001395 | 1.4 | 7.67E-07 |
| CRYBG1 | absent in melanoma 1 | NM_001624 | -0.9 | 7.68E-07 |
| HSP75 | TNF receptor-associated protein 1 | NM_016292 | -0.8 | 7.79E-07 |
| EPN3 | epsin 3 | NM_017957 | -0.9 | 8.04E-07 |
| PRAD1 | cyclin D1 | NM_053056 | -1.3 | 8.07E-07 |
| TOBL | transducer of ERBB2, 2 | NM_016272 | -0.8 | 8.30E-07 |
| Nrpn | kallikrein-related peptidase 8 | NM_144505 | -0.8 | 8.42E-07 |
| MGC138549 | microtubule-associated protein tau | NM_016835 | -0.9 | 8.61E-07 |
| IL22R | interleukin 22 receptor, alpha 1 | NM_021258 | -0.8 | 8.63E-07 |
| FLJ46310 | zinc finger protein 708 | NM_021269 | 0.8 | 8.65E-07 |
| Pam14 | Mof4 family associated protein 1 | NM_033296 | -0.9 | 8.68E-07 |
| SH3BP5 | SH3-domain binding protein 5 (BTK-associated) | NM_004844 | -1.0 | 8.71E-07 |
| CGI-25 | nitric oxide synthase interacting protein | NM_015953 | -0.8 | 8.75E-07 |
| RAF1 | v-raf-1 murine leukemia viral oncogene homolog 1 | NM_002880 | -0.9 | 8.91E-07 |
| PTGR1 | prostaglandin reductase 1 | NM_012212 | -0.9 | 8.95E-07 |
| M11S1 | cell cycle associated protein 1 | NM_005898 | -0.9 | 9.01E-07 |
| Ric8 | resistance to inhibitors of cholinesterase 8 homolog A (C. elegans) | NM_021932 | -1.2 | 9.03E-07 |
| MCUL1 | fumarate hydratase | NM_000143 | -0.8 | 9.04E-07 |
| AP2B1 | adaptor-related protein complex 2, beta 1 subunit | NM_001282 | -0.7 | 9.33E-07 |
| RNU12-2P | RNA, U12 small nuclear | NR_000041 | 1.0 | 9.33E-07 |
| SCD1 | stearoyl-CoA desaturase (delta-9-desaturase) | AF132203 | -1.4 | 9.53E-07 |
| AXSF | similar to growth arrest-specific 6; growth arrest-specific 6 | NM_000820 | -1.2 | 9.54E-07 |
