# Supplementary material for: Prospective Transcriptomic Pathway Analysis of Human Lymphatic Vascular Insufficiency: Identification and Validation of a Circulating Biomarker Panel
Source: PLoS One. 2012 Dec 18;7(12):e52021. doi: 10.1371/journal.pone.0052021 (PMC3525657; doi:10.1371/journal.pone.0052021)
Supplement: Table S2 — Secreted proteins of interest identified by microarray analysis. (DOC) [file pone.0052021.s002.doc]

**SUPPLEMENTAL MATERIAL**

**Table S2. Secreted Proteins of Interest**

| ADAM Metallopeptidase with Thrombospondin type 1 motif, 13 | Interferon, alpha 5 |
| --- | --- |
| Adrenomedullin | Interferon, alpha 8 |
| Alpha-1-B glycoprotein | Interferon, gamma |
| Alpha-2-HS glycoprotein | Interleukin 1 family, member 7 (zeta) |
| Angiopoietin 1 | Interleukin 1 family, member 9 |
| Angiopoietin 2 | Interleukin 1, alpha |
| Angiopoietin 2B | Interleukin 10 |
| Angiopoietin-like 7 | Interleukin 12B (lymphocyte maturation factor 2, p40) |
| APOBEC1 Complementation Factor | Interleukin 13 |
| Apolipoprotein A-I | Interleukin 17A |
| Apolipoprotein A-IV | Interleukin 17D |
| Apolipoprotein D | Interleukin 17F |
| Apolipoprotein F | Interleukin 18 binding protein |
| Apolipoprotein L | Interleukin 19 |
| Bactericidal/permeability-increasing protein | Interleukin 20 |
| Bone morphogenetic protein 10 | Interleukin 24 |
| Carcinoembryonic antigen-related cell adhesion molecule 1 | Interleukin 25 |
| Cardiotropin 1 | Interleukin 26 |
| Cathepsin B | Interleukin 28A (interferon, lambda 2) |
| Chemokine (C-C motif) ligand 25 | Interleukin 4 |
| Chemokine (C-X-C motif) ligand 17 | Interleukin 6 (interferon, beta 2) |
| Chorionic somatomammotropin hormone 1 (placental lactogen) | Interleukin 7 |
| Chromogranin A, parathyroid secretory protein 1 | Kallikrein-related peptidase 3, Prostrate-specific antigen |
| Chymotrypsinogen B2 | Lipoprotein, Lp(a) |
| Complement component 1, q subcomponent-like 1 | Pancreatic lipase-related protein 2 |
| Complement component 8, alpha polypeptide | Pentraxin 3 (pentraxin-related gene) |
| Complement factor H | Phospholipase A2, group sPLA2S IID |
| Cytokine-like 1 | Plasminogen |
| Epstein-Barr virus induced 3 | Pregnancy specific beta-1-glycoprotein 9 |
| Fibroblast growth factor 10 | Renin |
| Growth arrest-specific 1 | Serpin peptidase inhibitor, member 1 |
| Hepatocyte growth factor (hepapoietin A) | Transforming growth factor, beta |
| Immunoglobulin kappa variable 4-1 | Thrombopoietin |
| Inter-alpha (globulin) inhibitor H5-like | Tumor necrosis factor (ligand) superfamily, member 15 |
| Interferon, alpha 2 | Tumor necrosis factor superfamily, member 9 |
| Interferon, alpha 21 | Vascular endothelial growth factor A |
| Interferon, alpha 4 | VGL high density lipoprotein binding protein |
